# Clinical biomarkers and biosensors for early detection of metabolic dysfunction-associated steatohepatitis (MASH): a translational perspective

**DOI:** 10.3389/fbioe.2026.1748835

**Published:** 2026-01-23

**Authors:** Fareeha Arshad, Raja Chinnappan, Dieter C. Broering, Dimitri Aristotle Raptis, Tanveer Ahmad Mir, Mohammed Imran Khan, Ahmed Yaqinuddin

**Affiliations:** 1 College of Medicine, Alfaisal University, Riyadh, Saudi Arabia; 2 Tissue/Organ Bioengineering and BioMEMS Lab, Organ Transplant Centre of Excellence (TR&I Dpt), King Faisal Specialist Hospital and Research Centre, Riyadh, Saudi Arabia; 3 Organ Transplant Centre of Excellence, King Faisal Specialist Hospital and Research Centre, Riyadh, Saudi Arabia; 4 King Faisal Specialist Hospital and Research Center, Jeddah, Saudi Arabia

**Keywords:** biosensors, diagnostics, liver disease, MASH, NAFLD, NASH, point of care

## Abstract

Nonalcoholic steatohepatitis (NASH) or metabolic dysfunction-associated steatohepatitis (MASH) is a long-term chronic liver disease condition that stems from nonalcoholic fatty liver disease (NAFLD) and results from multiple factors, including lifestyle, metabolic dysfunction, and genetic predisposition. The increasing prevalence of NAFLD in the global population is expected to reach over 35% by 2030. It thus has become a significant public health concern because of its association with metabolic syndrome, cardiovascular diseases, diabetes mellitus, and hepatocellular carcinoma. Therefore, early diagnosis is crucial to avoid further liver disease complications and to provide early and effective patient care. Though there are diagnostic measures available for NASH/MASH detection, like biopsy and serological assays, these are mostly invasive and do not provide the complete picture of the liver condition. Point-of-care diagnostics like biosensors can help overcome these limitations by allowing for a rapid, inexpensive, and more straightforward diagnostic method that also aligns with the present global health needs. Moreover, integrating artificial intelligence and machine learning approaches for automated analysis alongside real-time cloud-based reporting and telehealth interfaces can potentially aid in expanding the utility of these systems into integrated diagnostic systems. Through this review, we aim to address the interplay of technological innovation, public health significance, and implementation barriers in advancing biosensor diagnostics for effective and reliable detection of NASH/MASH for better liver health.

## 1 Introduction

Nonalcoholic fatty liver disease (NAFLD) is a long-term, chronic liver disease condition that results from excessive eating habits and insulin resistance and is also influenced by genetic factors. Multiple diseases stem from NAFLD, including nonalcoholic fatty liver (NFL), metabolic dysfunction-associated steatohepatitis (MASH), nonalcoholic steatohepatitis (NASH), cirrhosis, and others (Guo Z. et al., 2025). The occurrence of metabolic dysfunction-associated steatotic liver disease (MASLD) is expected to increase from ∼33% in 2020 to over 41% in 2050, which means there will be more than 120 million cases of MASLD among the US adults alone by 2050. Because of its ever-increasing global prevalence in recent years and its association with metabolic syndrome and diabetes mellitus (Godoy-Matos et al., 2020), cardiovascular diseases (Chong et al., 2025), chronic kidney diseases, along with cancers like hepatocellular carcinoma (Shah et al., 2023), NAFLD has become a significant public health concern. In a study by Estes et al., the researchers confirmed that the prevalence of NAFLD will increase to over ∼35% by 2030 (Estes et al., 2018). Moreover, scientists also speculate that about 38% of the general population is affected by NAFLD, which will increase up to ∼56% by 2040 (Nguyen et al., 2022; Younossi et al., 2023). In addition, over 5% of the population experiences some progressive form of NAFLD, that is, NASH, also known as MASH, which causes multiple liver-associated clinical disorders (Le et al., 2025). This is particularly more common among adult males, especially within the high-income western countries, and has also rapidly increased in central and middle Asia, and also within the North African countries since the mid-1990s (Dong et al., 2024). Recent meta analyses also indicate that the Middle Eastern and North African countries (MENA) and the Asian countries have among the highest NAFLD/NASH burdens worldwide with about one-third adults being affected with fatty liver diseases, which includes, 36.5% from the MENA, 33.8% in South Asia, 33.1% in South East Asia, and ∼30% in East Asia thus implying a large at risk pool for NASH (Younossi et al., 2023). Furthermore, ∼6% of the general population in these regions are already affected by NASH/MASH, with 5.85% in MENA and ∼5.4% in South and Southeast Asia (Younossi et al., 2023). In addition, Asia alone accounts for ∼57% of global metabolic-associated steatotic liver disease cases (Sarkoohi et al., 2025).

**FIGURE 1:**
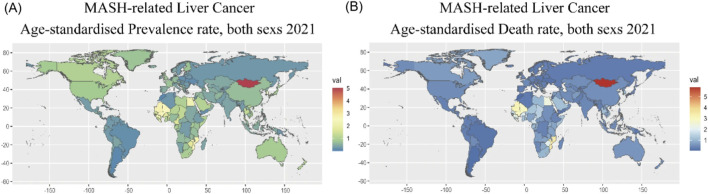
Global burden of NASH **(A)** Prevalence rate **(B)** Death rate. Adapted with permission (Guo Z. et al., 2025) 2025, Nature.

The effect of NASH/MASH is influenced by multiple factors, including the metabolic age, insulin resistance, presence of type 2 diabetes, genetic factors, and also external environmental factors (Tapper et al., 2023). Also, the interdependence of insulin resistance and type 2 diabetes amplifies the occurrence of NASH/MASH, which also further adds to additional health risks like advanced fibrosis, liver mortality, and cancers (Golabi et al., 2023). The prevalence of developing MASH-related hepatocarcinoma and death rate is shown in Figure 1.

Development of MASH occurs multifactorially; that is, with excessive fatty acids in the liver, the liver becomes more prone to injuries by multiple factors, including excessive reactive oxygen species (ROS) production, fatty acid and alcohol oxidation alongside obesity as adipose tissues release inflammatory markers like leptin, interleukin-6 (IL-6), and tumor necrosis factors (TNF) (Girish V, 2025). Under such circumstances, the hepatocytes transform into balloon-like cells with cytoskeletal aggregation, resulting in apoptosis and necrosis (Xian et al., 2021). Furthermore, with insulin resistance, sinusoidal collagen deposition occurs, which leads to the further progression of MASH and progressive fibrosis (Tiniakos et al., 2023). In most cases, liver biopsy is indispensable for confirmatory diagnosis of NASH/MASH. Upon confirmation of MASH, patients are still required to undergo routine non-invasive assessments with hepatologists or gastroenterologists for advanced fibrosis based on clinical development. Therefore, non-invasive diagnostic tools are needed for rapid NASH/MASH screening and monitoring. Using such detection systems will help in the easy recognition of at-risk patients and quick referral for liver care, as well as remove the burden of invasive methods.

Several biomarker tests, like enhanced liver fibrosis, provide an idea of advanced fibrosis and overall liver health. Another such test is the FAST score that provides FibroScan and AST to assess NASH progress (Al Hashim et al., 2025), which can be combined with transient elastography that non-invasively confirms active MASH and fibrosis progress in patients (Newsome et al., 2020). Enhanced liver fibrosis score that measures hyaluronic acid, metalloproteinase 1, and procollagen III N-terminal peptide also provides valuable insights for liver health (Saarinen et al., 2023). Though these diagnostic methods are the gold standard for confirmation of NASH/MASH, they require comprehensive sample preparation steps, prolonged processing durations, and sophisticated instruments that can prolong diagnostic time and further treatment (Khalifezadeh et al., 2026). Therefore, alternative diagnostic options like biosensors are promising alternatives that can potentially perform highly sensitive, selective, specific, and rapid detection of NASH/MASH-associated biomarkers. Biosensors are device systems that record the interaction between the analyte and its corresponding bioreceptor and convert it into a quantifiable signal response. They are minimally invasive, cost-effective point-of-care (POC) systems for diagnostics that are also highly selective based on the bioreceptor that targets the analyte explicitly and provides a highly sensitive signal response that can detect trace concentrations of the target in a given sample and are promising diagnostic platforms that can potentially be applied to detect and assess the progress of NASH/MASH in patients.

Through the present paper, we provide an extensive overview of the application of biosensors for detecting and monitoring NASH/MASH biomarkers, pushing the boundaries of traditional diagnostic methods. Though there have been a few recent articles that discuss the application of electrochemical biosensors for sepsis detection in liver cirrhosis (Kaur and Singh, 2025) and liver cancers (Du and Tao, 2025), or fluorescent biosensors for precision detection of liver diseases (Wu et al., 2025), and exosome biosensors for detection of liver cancers (Vafadar et al., 2025); however, to the best of our knowledge, no comprehensive published literature discusses the promising potential of biosensors for NASH/MASH monitoring. Therefore, to better understand the working of various biosensors and advances in sensor development for liver disease diagnostic applications, we have an in-depth understanding of recent progress in this field. We first provided a brief overview of the pathophysiology of NASH/MASH, followed by a detailed discussion on the current diagnostic options available and the need for a biosensing approach. We then discussed the different types of biosensors that can potentially be applied to detect different biomarkers associated with NASH/MASH. Subsequently, we highlighted the current advances and proof-of-concept studies, and also the translational and clinical considerations related to such biosensors. Finally, we provided future perspectives that will aid in bridging the gap between biochemical pathways and clinical and analytical applications for rapid prognosis and diagnosis of liver-associated diseases.

## 2 Pathophysiology of NASH/MASH

**FIGURE 2:**
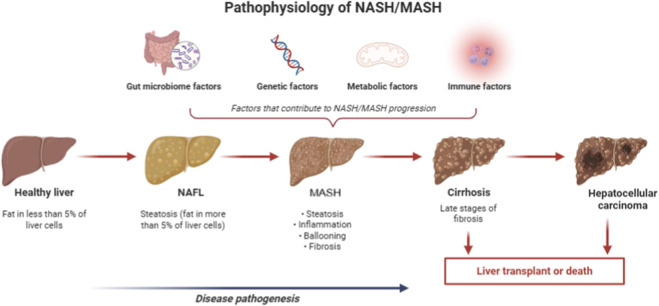
Pathophysiology of NASH/MASH and disease progression.

The onset of steatosis defines the onset of NASH/MASH, that is, the presence of over 5% liver fat followed by oxidative stress and injury (Zhu et al., 2023), as shown in Figure 2. The liver removes these fatty acids through beta oxidation or by moving them as very low-density lipoproteins. When adipocytes are heavily taxed, then non-esterified fatty acids are released into plasma, causing elevated lipolysis (Wattacheril et al., 2018).

An increase in liver triglycerides can help to process toxic lipids into triglyceride-enriched lipid droplets and, therefore, potentially serves as a self-preservation strategy to regulate the excess energy in hepatic tissues (Parola and Pinzani, 2024). Thus, the fat type that usually gets stored in the liver, neutral fat or triglycerides, differs from the toxic fat that causes those results in NASH/MASH (Loomba et al., 2021). This series of metabolic pathways causes mitochondrial dysfunction that inhibits oxidative phosphorylation and ATP synthesis within the cells and therefore impairs their structure and functionalities, subsequently leading to the accumulation of ROS followed by damage to the endoplasmic reticulum (ER) and lipid peroxidation pathways (Loomba et al., 2021). Consequently, this liver cell cytotoxicity also causes liver tissue malfunction, oxidative and ER stress, partial autophagy, and imbalances in gut microbiome, leading to hepatocyte inflammation (Kolodziejczyk et al., 2019).

Other factors like genetic conditions, including PNPLA3 polymorphism, also further exacerbate the progression of NASH/MASH (Targher et al., 2024; Li Y. et al., 2024). Overexpression of tumor necrosis factor (TNF) α occurs primarily in the hepatocytes of overweight patients, thereby continually activating IκB kinase β that regulates the addition of a phosphoryl group in the insulin receptor substrates 1 and 2. Genetic and epigenetic-associated mitochondrial malfunction also happens in NASH/MASH individuals, characterized by decreased mitochondrial DNA, reduced ATP, leakage of lysosomal enzymes that in turn activate NF-κB and TNF-α, resulting in cell apoptosis (Li et al., 2008; Tantu et al., 2025). Hence, liver cell injury, damage, and death are the result of the metabolic pathways that lead towards fibrosis and liver cirrhosis.

## 3 Current diagnostic landscape

The most commonly effective and accurate gold standard method to determine the intensity of NASH/MASH is liver biopsy, which is an invasive technique (Eslam et al., 2020; Rizzo et al., 2023; Sergi, 2024). Apart from histopathological assessment, immunocytochemical and electron microscopic tests, microbiological and biochemical analyses can also be performed on the liver tissues derived during the biopsy (Sergi, 2024). Lobular inflammation, steatosis, and hepatocyte ballooning are the most common markers in biopsy results during NASH/MASH, and the NAFLD activity score is commonly applied for systematically evaluating biopsy results. Multiple non-invasive tests are also available, including serological tests and imaging techniques like ultrasonography, computed tomography, and magnetic resonance imaging, to assess the onset and progression of NASH/MASH (Table 1). Amongst these, ultrasonography is the most commonly used as it is inexpensive and readily available (Sheka et al., 2020). Other serological tests include AST to platelet ratio index score (APRI) and fibrosis-4 calculator that help to predict and assess NASH/MASH. Other commercial fibrosis marker tests including enhanced liver fibrosis and FibroTest are also useful to predict and thus exclude the cases associated with advanced fibrosis. Radiological methods that use ultrasound transient elastography like FibroScan, magnetic resonance elastography, and acoustic radiation force impulse imaging are also reliable in prognosing and diagnosing NASH/MASH (John, 2023). However, though these methods are commonly used in diagnosing liver diseases, there are a few limitations that still need to be overcome.

**TABLE 1:** Comparison of current diagnostic tools for NASH/MASH analysis.

Method	Advantages	Limitations	Clinical use
Biopsy	Accurate diagnosis and staging; provide detailed histological information	Invasive; potential sampling variability; high costs; resource-intensive	Diagnostic confirmation, disease staging; clinical intervention
Immunocytochemical tests	Detect specific protein markers like cytokeratin-18 at cellular level; helpful in identifying hepatocyte injury and fibrosis activity	Limited sensitivity and specificity; semi-quantitative; requires tissue or cell samples; not suitable for large-scale screening	Assess cellular changes and biomarker expression
Electron microscopic tests	Ultra-structural evaluation of hepatocytes, mitochondria, and lipid droplets; high-resolution visualization of subcellular changes	Technically demanding; costly; limited availability; not routinely applicable to clinical practice	Elucidate disease mechanisms and validate histopathological findings
Microbiological tests	Identify alterations in gut microbiota composition linked to NASH pathogenesis	Indirect diagnostic relevance; variability between individuals; lack of standardization; complex data interpretation	Research and exploratory biomarker studies; adjunctive evaluation of metabolic dysfunction
Serological tests	Non-invasive; panels such as FIB-4, NAFLD fibrosis score, and ELF test estimate fibrosis and inflammation; useful for risk stratification	Limited accuracy for intermediate fibrosis stages; may be affected by age, BMI, and comorbid conditions	Screening, fibrosis risk assessment, and monitoring of disease progression
Acoustic radiation force impulse imaging	Real-time elastography using ultrasound; non-invasive; portable	Operator-dependent; limited accuracy in early fibrosis; affected by inflammation and steatosis	Can be potentially applied for bedside evaluation of liver stiffness and fibrosis staging; screening in clinical settings

For starters, biopsies have been shown to have some sampling errors, and to reduce the error, adequate tissue must be collected, about 1.5–1.6 cm in length or longer, to accurately evaluate the disease stage [53, 54]. In addition, inter- and intra-observer variation also poses a significant issue during pathological confirmation of NASH/MASH, and therefore, more stringent training and measures are needed to overcome these inconsistencies (Gawrieh et al., 2011). More importantly, because of the invasive procedures, there are additional risks and complications associated with discomfort and even mortality that have been reported in worst-case scenarios (Van Der Poorten et al., 2006; Sumida et al., 2014). In addition, only a limited number of patients display all the symptoms, and thus, there is no unified diagnostic measure that can confirm NASH/MASH in them. Therefore, other diagnostic platforms like biosensors can be potentially explored towards NASH/MASH diagnosis and monitoring.

## 4 Need for biosensing approaches

Biosensors are a promising technology, especially in the field of medicine, engineering, and healthcare for diagnostic and analytical applications. It integrates biological sensing units that include aptamers, antibodies, and enzymes, along with a physicochemical detection system that causes the conversion of biochemical activities to measurable and quantifiable signals (Manoharan Nair Sudha Kumari and Thankappan Suryabai, 2024). Owing to the favorable advantages compared to conventional analytical techniques, including easy and rapid detection, enhanced sensitivity, easy portability, user friendliness, no need for skilled technicians or bulky equipment, and the ability to carry out real-time POC analysis, biosensors facilitate direct and rapid clinical decisions for further ease in healthcare management (Arshad et al., 2022).

Biosensors detect any target analyte based on the specific interaction between the biomolecule and the corresponding bioreceptor to evaluate the target concentration. In biosensors, a bioreceptor is immobilized over a convenient substrate via physical adsorption, covalent, or non-covalent binding (Huang et al., 2023). The interaction between the biological interfaces, that is, the bound bioreceptor and the target analyte, via adhesion or affinity, results in the evaluation based on the changes in mass, optical properties, electrical aspects, and others (Ramesh et al., 2023). Based on the recorded modifications, a physicochemical transducer converts to a quantifiable signal like electrical, optical, or sound wave signals, which are then understood with the help of a signal amplifier (Abdel-Karim, 2024). The signal processing is performed with the help of an amplifier, which acts as a bridge between the sample signal and reference signals. Upon receiving the digital signals, the microprocessor processes and shows the results on the screen for the user to understand. Thus, the bioreceptor, transducer, and the signal amplifier and processor interfaces work seamlessly as a unit in biosensors to give sensitive, selective, and rapid results (Khattab, 2024). Biosensors that can detect pathophysiological changes, especially at molecular levels, such as oxidative stress markers, inflammatory cytokines like TNF-α and IL-6, and lipid peroxidation products, can potentially allow for the identification of subclinical steatohepatitis before any fibrogenic transformation takes place. For instance, electrochemical or optical biosensors that can be integrated into wearable or POC systems and continuously assess multiple biomarkers like glucose levels, triglycerides, circulating miRNAs associated with hepatic inflammation and injury can serve as promising tools in NASH/MASH. Such systems would allow clinicians to detect early biochemical changes indicative of treatment response or relapse, thus facilitating timely therapeutic adjustments. Given this need, various biosensor platforms have been developed, as summarized in the next sections.

## 5 Types of biosensors relevant to NASH/MASH

Multiple types of biosensors can be potentially applied for NASH/MASH detection and monitoring for early medical intervention, as discussed in Table 2.

**TABLE 2:** Broad classification of biosensors and their advantages and limitations.

Biosensor type	Target analyte	Detection principle	LOD	Linear range	Sample matrix type	Advantages	Limitations	Ref.
Electrochemical biosensors	ALT, AST, glucose, uric acid	Enzyme-catalyzed redox reactions producing measurable current or potential response	2.97 U/L (for ALT)	25–700 U/L (for ALT)	Plasma samples	• High sensitivity • Rapid response • Compatible with miniaturization and POC testing	• Possible enzyme instability • Interference from biological matrices • Requires calibration	Samy et al. (2023), Sun et al. (2023), Alatzoglou et al. (2024)
Optical biosensors	CK-18, adiponectin, IL-6, TNF-α, ROS	Changes in absorbance, fluorescence, or plasmon resonance upon target binding	3.0 × 10^−16^ g/mL (for glucose); 0.13 μg/mL (for TNF-α)	10^−15^ to 10^−6^ g/mL (for glucose); 100–1,500 ng/mL (for TNF-α)	Serum samples	• Label-free or label-based detection • Real-time monitoring • High specificity	• Complex optical setups • Sensitive to environmental fluctuations • May require signal amplification	Rajeev et al. (2018), Kaur et al. (2022), Kim et al. (2022), Lin and Tan (2023), Sojdeh et al. (2024)
CRISPR/Cas-based biosensors	Circulating nucleic acids (miR-122, miR-34a, miR-192), inflammation-related genes	Target recognition by guide RNA-Cas complex leading to collateral cleavage and fluorescent or colorimetric signal	-	-	Buffer samples	• High specificity • Programmable detection of genetic markers • Amenable to low-cost platforms	• Requires nucleic acid extraction • Limited validation in clinical samples • Reagent storage stability issues	Zhuang et al. (2022), Kumaran et al. (2023), Zhou et al. (2024)
Nanomaterial-based biosensors	Oxidative stress markers, lipid metabolites, and liver enzyme substrates	Signal amplification via catalytic or plasmonic activity of nanomaterials (like AuNPs, CeO_2_, CNTs)	10 ng/mL (for 8-OHdG)	10 μg/mL to 100 μg/mL (for 8-OHdG)	Saliva and urine samples	• Enhanced sensitivity and stability • Adaptable to multiple analytes • Cost-effective fabrication	• Complex synthesis and reproducibility issues • Potential cytotoxicity of nanomaterials	Sondhi et al. (2020), Bai and Li (2023), Zhu et al. (2024)
Microfluidic-based biosensors	ALT, AST, CK-18, glucose, lactate, cytokines	Integrated microchannels enabling multiplexed biochemical assays with minimal sample volume	0–200 μM (for glucose); 1 to 10,000 pg mL^–1^ (for cytokines)	30 µM (for glucose); 0.46–1.36 pg mL^–1^ (for cytokines)	Buffer	• High throughput • Minimal reagent use • Suitable for multi-analyte detection	• Fabrication complexity • Potential biofouling • Limited clinical standardization	Gao et al. (2021), Chinnappan et al. (2023), Lokar et al. (2023), Shi et al. (2023)

### 5.1 Electrochemical biosensors

Electrochemical biosensors are analytical systems that convert biological interactions, like enzyme-substrate or antigen-antibody interactions, into corresponding measurable electrical signals (Cho et al., 2020). Such biosensors, owing to their ability to rapidly detect biomolecules even at very low concentrations with high reproducibility in complex matrices, are promising for developing miniaturized POC devices for NASH/MASH biomarker detection (Arshad et al., 2024).

#### 5.1.1 ALT detection

Several electrochemical biosensors have been studied and explored to detect multiple biomarkers associated with NASH/MASH, including ALT/AST activity, lipid peroxidation markers, free fatty acids, and 2-hydroxyglutarate, among others. For instance, in a recent study by Wang et al., a real-time biosensor was developed for ALT detection that used functionalized crystal microcavities functionalized with stearic acid and integrated with a whispering gallery mode laser (Wang J. et al., 2025). The presence of liquid crystal microcavities improved the biosensing performance, and the results showed a linear correlation within the range of 1–240 U/L and a low limit of detection (LOD) of 0.67 U/L, and thus is promising towards early detection of liver diseases. Similarly, in another recent study, graphene oxide and multiwalled carbon nanotubes hybrid along with gold nanoparticles (AuNP) and glutamate oxidase was integrated with surface plasmon resonance (SPR) based fiber biosensors for highly sensitive ALT detection within the linear range of 0–1,000 U/L and demonstrated a low LOD of 4.84 U/L and could perform the detection within 20 min with high sensitivity and selectivity (Singh et al., 2023). In a separate study by Muratore and colleagues, silicon nanowire field-effect transistor sensors were developed that could perform real-time monitoring of ALT over a wide range (Muratore et al., 2023). The team proposed a dual-mode biosensor that could perform the ALT detection via spectrophotometric and electrochemical methods by combining ALT-assisted pyruvate synthesis and ferricyanide reduction, and thus enabling a dual-mode response for rapid ALT detection.

#### 5.1.2 AST detection

Recently, several studies have been conducted to explore reliable biosensors for monitoring AST activity. In a recent study by Mruga et al, an amperometric biosensor was developed based on a platinum disk electrode for assessing AST levels in various biological fluids (Mruga et al., 2024). To confirm sensor efficiency, substrates such as aspartate, α-ketoglutarate, and pyridoxal phosphate coenzymes were used in reliable concentrations, and a semipermeable membrane based on polyphenylenediamine was used to improve the sensor selectivity in the presence of interfering molecules. The researchers confirmed that a good analytical performance in the proposed sensor was observed with a linear range between 1 and 110 U/L, a LOD of 1 U/L, and thus could also be applied in real sample analysis with high reproducibility. In another study by Samy and colleagues, an electrochemical microelectrode biosensor was developed using molecularly imprinted pyruvate oxidase enzyme by employing 4-aminophenol, platinum nanoparticles, and 4-aminoantipyrine along with sodium pyruvate that functioned as the electrochemical interface (Samy et al., 2023). The proposed biosensor displayed a reasonable linear range between 25 and 700 U/L and LOD of ∼3 U/L and could perform the detection with selectivity and reproducibility in plasma samples. The significant advantage of the biosensor was the promising approach towards AST detection without requiring any extensive sample preparation, the ability to be integrated into portable POC diagnostics for continuous monitoring of the liver health in disease individuals without relying on any invasive procedures.

#### 5.1.3 Lipid peroxidation markers detection

**FIGURE 3:**
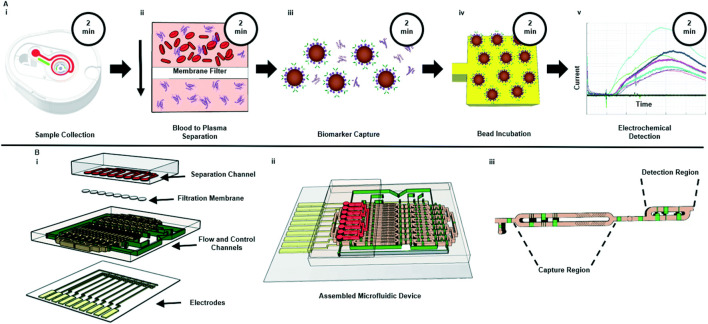
Schematic of **(A)** Assay workflow of the POC platform workflow and associated durations from sample collection to electrochemical detection. **(B)** Microfluidic device with an exploded view of the multi-layer microfluidic system. Reproduce with permission (Kikkeri et al., 2022) 2022, RSC.

As discussed in the previous section, lipid peroxidation markers serve as crucial factors in understanding and evaluating the progression of NASH/MASH. A few studies have been conducted in the recent decade towards developing such reliable biosensors that can be used to assess the lipid peroxidation markers. In a study by Attig and colleagues, a porphyrin-modified magnetic graphene oxide (TCPP-MGO) functionalized screen-printed carbon electrode (SPCE) based electrochemical sensor was developed that could perform highly sensitive and selective detection of malondialdehyde (MDA), which functions as an essential marker of oxidative damage (Ben Attig et al., 2023). Upon the integration of TCPP with MGO, the magnetic properties of the resultant material were applied for sample preprocessing, which aids in the selective detection of the target analyte. In addition, the electrochemical property of the SPCE was further enhanced by derivatizing MDA with diaminonaphthalene. Hence, the resultant sensor displayed a rapid detection response over a broad linear range between 0.01 and 100 µM with an LOD of 0.010 µM. Furthermore, the team confirmed that the proposed sensor could perform reliable MDA analyte detection even in real serum samples with high reproducibility. In another study by Kikkeri et al., an automatic electrochemical biosensor was developed that could perform rapid detection of interleukin-6 from whole blood samples (Kikkeri et al., 2022) in less than 30 min, as shown in Figure 3. The system integrated a microneedle blood sampling interface along with a membrane-based plasma filtration over a bead-based electrochemical immunoassay. The resultant biosensor displayed a high separation efficiency with minimal nonspecific binding over the filtration membrane under various experimental conditions. Therefore, the proposed platform is a promising approach that can be applied in sample preparation, especially under POC settings in continuous monitoring of NASH/MASH-affected patients without requiring extensive sample acquisition or preparation methodologies and invasive techniques.

#### 5.1.4 Free fatty acids detection

Free fatty acids detection is also crucial in confirming NASH/MASH status in diseased individuals. A few studies have been conducted in recent years that explore the prospects of developing sensitive and portable biosensors for fatty acid detection. For instance, in a study by Giaretta et al., a conductive poly (3,4-ethylenedioxythiophene): polystyrenesulfonate (PEDOT:PSS) was incorporated into a thread-like structure along with horseradish peroxidase and lipoxygenase to develop a prototype of wearable biosensors or biomarker detection (Giaretta et al., 2024). The proposed sensor could selectively confirm the presence of linoleic acid within a wide linear range of 161 nM to 16.1 µM. It can be applied towards the sensitive detection of fatty acids. Similarly, in another study by Wang and colleagues, another wearable non-invasive biosensor prototype was developed that could detect the target analyte with high sensitivity and selectivity even in sweat samples (Wang et al., 2022). The sensor was comprised of graphene electrodes decorated with metabolite-specific antibody-based molecularly imprinted polymers and redox-active reporter nanoparticles. The system was combined with iontophoresis-based sweat initiation structures, microfluidics for sweat processing, and signal generation. The resultant biosensor could perform real-time detection and continuous monitoring of various target molecules from sweat. It can therefore be applied towards developing wearable biosensors for early identification and intervention in NASH/MASH amongst the affected individuals. Smart and coworkers also developed an electrochemical biosensor that could detect free fatty acids from a variety of sample matrices. They used screen-printed carbon electrodes that were functionalized with cobalt pthalocyanine and fabricated an amperometric biosensor. The fabricated biosensor showed a wide linear range between 0.2 and 10 μM and 0.2–10 µM with a LOD of 24 nM and 100 nM for linoleic acid and α-linoleic acid, respectively, and therefore holds promising applicability in non-invasively detecting free fatty acid biomarkers in NASH/MASH-affected patients.

#### 5.1.5 D-2-hydroxyglutarate (D-2-HG) detection

D-2-HG is an oncometabolite in cancers that are associated with isocitrate dehydrogenase (IDH) mutations and are essential in cell signaling and immune response, and therefore serves as a crucial biomarker in the diagnosis and prognosis of cancers, including liver cancers that occur as a consequence of NASH/MASH (Lin et al., 2024). It is primarily a byproduct of 4-hydroxybutyrate to succinic acid in the presence of hydroxyacid-oxoacid-transhydrogenase (Toplak et al., 2019). Usually, D-2-HG undergoes oxidation to give α-ketoglutarate (α-KG) in the presence of D-2-HG dehydrogenase. However, when the enzymes associated with D-2-HG metabolism undergo mutations, there is about a 100-fold elevation of D-2-HG in the body, resulting in D-2-hydroxyglutaric aciduria (Struys, 2006). Therefore, elevated D-2-HG not only serves as a marker for IDH-mutated conditions but is also crucial in the diagnosis of D-2-hydroxyglutaric aciduria and cancers.

Recently, multiple studies have been carried out to assess the applications of biosensors in D-2-HG detection that can be potentially applied in liver disease monitoring. For instance, in a recent study by Hou et al., D-2-HG dehydrogenase (D-2-HG-DH) was used, which allows direct electronic transfer with chemical electronic mediators like methylene blue (MB) without requiring any extra coenzymes (Wu et al., 2022). The D-2-HG-DH was immobilized along with MB over Ti_3_C_2_ MXene and was subsequently coated onto a gold screen-printed electrode to fabricate a portable and miniaturized biosensor. Chronoamperometric response was recorded by the proposed biosensor, which confirmed a wide linear range between 0.5 and 120 µM and a low LOD of 0.1 µM. Furthermore, this portable biosensor demonstrated up to ∼72.5% of its original performance even after a month of storage and gave high reproducibility even in serum and urine samples. Thus, the electrochemical biosensor could be applied towards non-invasive detection of NASH/MASH biomarkers with high selectivity and sensitivity.

#### 5.1.6 CK-18 fragments

Cytokeratin-18 (CK-18) is the main intermediate filament in hepatocytes and is released upon hepatocyte death (Lee et al., 2020). Because MASH involves hepatocyte injury and apoptosis, CK-18 fragments like M30/M65 have been extensively studied as circulating biomarkers (Kawanaka et al., 2024). Electrochemical immunosensors can potentially convert CK-18 antibody-antigen binding into an electrical signal, enabling rapid, label-free quantitation (Yaman et al., 2024). Integration of nanomaterials can significantly enhance the performance of the resultant biosensors. For instance, in a recent study by Zhao et al., an electrochemical biosensor was proposed using a chemically modified electrode for CK-18 detection (Zhao et al., 2022). Bismuth sulfide nanocrystals were used to alter the working electrode, which was then further functionalized with CK18 antibodies (Zhao et al., 2022). The team confirmed that the modified electrode provided reliable charge transfer capability and could detect CK-19 antigen protein with a low LOD of 1.87 fM and a wide linear range of 1–1,000 pg/mL within 30 s. Thus, such portable electrochemical platforms can be potentially integrated into point-of-care panels for early MASH screening. However, clinical implementation will require rigorous validation including testing these sensors in patient cohorts to determine how CK-18 sensing can complement other biomarkers to improve diagnostic accuracy and prognostic value of the resultant biosensors.

#### 5.1.7 miRNAs

As discussed in the previous section, circulating microRNAs have emerged as highly promising non-invasive biomarkers for fatty liver disease. In particular, hepatocyte-enriched miR-122 is consistently elevated in the plasma during liver injury, reflecting hepatocellular damage with high specificity and sensitivity (Roychoudhury et al., 2024). Other miRNAs such as miR-34a, miR-21, and miR-192 also correlate with steatosis and inflammation in steatohepatitis (Ragab et al., 2023). Electrochemical biosensors provide an attractive platform for miRNA detection. By translating the hybridization of target miRNAs with complementary probes into electrical signals, they enable rapid, label-free analysis with high sensitivity (Hunt and Slaughter, 2025). Moreover, such sensors are inherently portable, low-cost, and require minimal sample preparation, in contrast to PCR or sequencing. For instance, in a recent study by Primpray et al., a disposable screen-printed electrode modified with reduced graphene oxide and molybdenum disulfide was developed that could perform rapid detection of miRNA-34a (Primpray et al., 2025). The resultant biosensor could detect miRNA with a high sensitivity of 66 pM within a wide linear range of 0.1–1,000 nM. The team also confirmed high selectivity of the electrochemical biosensor for miRNA detection in serum samples, and thus displayed their promising potential. In another separate study by Nagdeve and colleagues, an electrochemical biosensor was developed for miRNA-31 detection (Nagdeve et al., 2025). The team modified a glassy carbon electrode with graphene to improve the selectivity and the sensitivity of the sensor. The fabricated system could detect miRNA at concentrations as low as 70 pg/mL in buffer and 700 pg/mL in serum samples, thus displaying exceptional performance towards diagnostic applications. Another similar electrochemical biosensor was developed for the detection of miRNA-21 (Guo J. et al., 2025). The sensor comprised target recycling amplification and non-hybridization chain reactions that aided in the signal amplification. Thus, the sensor could detect the miRNA-21 at concentrations as low as 0.8 fM within a wide linear range of 1 fM to 10 nM with high reproducibility and selectivity. Therefore, electrochemical miRNA biosensors hold significant promise for early, minimally invasive MASH diagnosis.

### 5.2 Optical biosensors

Optical biosensors work based on the signals produced by interacting biomolecules with light to give a quantifiable response about the target molecule. Optical biosensors rely on a bioreceptor that selectively interacts with the target molecule, providing a biochemical response that triggers changes in the optical property, like refractive index or modulation in the light intensity, which is converted to an optical signal by a transducer (Akgönüllü and Denizli, 2022). Thus, optical biosensors, because of their advantages like the possibility of real-time and label-free detection, enhanced sensitivity and selectivity, and inexpensive fabrication process compared to other conventional diagnostic methods, can be applied to non-invasive detection of NASH/MASH-related biomarkers.

#### 5.2.1 Circulating cytokines detection

**FIGURE 4:**
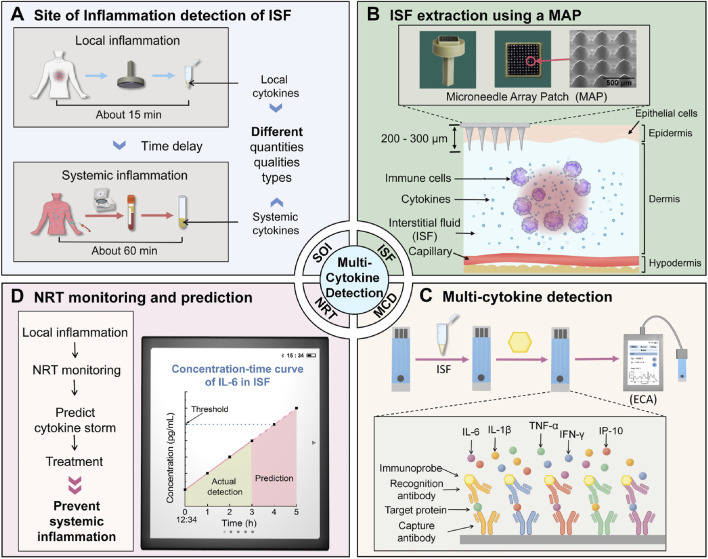
Immunosensor for detection of multiple proinflammatory cytokines. **(A)** Compared to blood, interstitial fluid is a superior body fluid (ISF) for near real-time (NRT) detection and site-of-inflammation sampling of cytokines. **(B)** 200–300 μm microneedle array patch is used to extract dermal ISF. **(C)** Five proinflammatory cytokines: IL-6, IL-1β, TNF-α, IFN-γ, IP-10 in ISF are detected by a printed carbon electrode and measured by a handheld electrochemical analyzer. **(D)** NRT detection results are imported into analysis and prediction software to monitor and guide clinical treatment of systemic inflammation and ensuing cytokine storms. Reproduced with permission (Li T. et al., 2024) 2024, Elsevier.

As discussed in the previous sections, circulating cytokines are crucial markers for liver inflammation and damage in NASH/MASH. Increased levels of pro-inflammatory cytokines play a part in liver injury, fibrosis, and the onset of severe conditions like cirrhosis. Therefore, the rapid diagnosis of these markers is pivotal for monitoring and treating liver-associated chronic disease conditions. Multiple studies have been carried out in recent years towards developing highly sensitive and selective detection of cytokines in a variety of sample matrices. For instance, in a study by Chen and colleagues, a quantum dot-based fluorescent immunosensor was developed to detect cytokines in serum (Chen et al., 2025). The team used carboxyl-capped CdTe quantum dots as fluorescent labels that were linked with lysine-rich biotinylated peptides to enhance fluorescence response, which was further functionalized with antibodies to develop fluorescent detection probes. The probes were then integrated over a fluorescence-linked immunosorbent assay for the detection of growth differentiation factor-15 (GDF-15) over a broad linear range of 6 and 1,600 pg/mL and a low LOD of 0.98 pg/mL with high reproducibility even in human serum samples. Therefore, the system can be potentially applied towards rapid diagnosis of NASH/MASH cytokine biomarkers. In another similar study by Li and coworkers, a near real-time and site-of-inflammation immunosensor was developed with ZIF-8 as the signal amplifier that could simultaneously detect multiple cytokines, including IL-6, IFN-γ, IL-1β, TNF-α, and IP-10 in as little as 5 µL of dermal interstitial fluid (Li T. et al., 2024), as shown in Figure 4. A microneedle array patch and a trifurcated nozzle pump were used to draw the interstitial fluid, which was then transferred to the real-time biosensor. The proposed sensor displayed a low LOD of ∼5.76 pg/mL with a broad linear range of 5.76–20 ng/mL and therefore can be applied towards detecting biomarkers even from interstitial fluids. In yet another study by Deng and colleagues, another multiplex optical fibre-based fluorescent biosensor was proposed that could perform simultaneous detection of IL-1β, IL-6, and TNF-α within a wide linear range of 12.5–200 pg/mL and a low LOD of 12.5 pg/mL. The biosensor was fabricated such that a streptavidin-modified fibre surface was first developed, followed by immobilizing biotinylated capture antibodies. Subsequently, the BSA solution was drop-cast to remove any nonspecific interaction with fluorescent magnetic beads. Thus, three distinct fluorescent magnetic beads comprising varying activation wavelengths were used to detect the 3 different cytokine targets simultaneously and hence also hold promising potential in the rapid diagnosis of multiple biomarkers within the same sample.

#### 5.2.2 Exosomes detection

Exosomes help carry out intercellular communications and perform pivotal functions towards developing multiple disease conditions, including NAFLDs, and hence serve as a potential biomarker for early diagnosis of NASH/MASH (Ding et al., 2025). Under cancerous conditions, more extracellular vesicles containing exosomes are released as compared to regular conditions and thus prove to be superior biomarkers for the disease prognosis and diagnosis (Ding et al., 2025). Multiple studies have been carried out in recent years to explore different biosensors for exosomal detection. For instance, in a recent study, a nanophotonic perfect absorber that operated within the tetrahertz range was used towards developing a microwave imaging, label-free biosensor for exosomes detection (Hamza et al., 2025). The biosensor included a silver resonator along with a nickel ground plane fixed over a silicon dioxide substrate containing intricate geometries that allowed for several resonance modes, permitting uninterrupted broadband adsorption, which gave a stronger signal response in the presence of tumour-causing exosomes than the normal exosomes based on their differences in molecular compositions. The researchers confirmed that the biosensor prototype is very promising in the rapid and non-invasive detection of exosomes. In another study by Barros et al., optical fiber tweezers were used biophotonic tools to develop a novel sensing method called the Hilbert Phae Slope that could easily distinguish between two different extracellular nanovesicles giving rise label free biosensing method that could be applied as a single molecule detection strategy (Barros and Cunha, 2024) and, therefore, is a powerful diagnostic device that can be applied for multiplexed detection of NASH/MASH biomarkers.

### 5.3 Nanomaterial-based biosensors

Nanomaterials, because of their nanoscale size, have versatile properties, including a large surface-to-volume ratio that permits strong nanomaterial interaction with the surrounding environment and hence also demonstrates high catalytic and absorptive properties (Yoon et al., 2020; Wang B. et al., 2025; Liu et al., 2025). In addition, they exhibit enhanced electronic conductivity, good mechanical and chemical activities, superior diffusivity, and are biocompatible (Cavalcante et al., 2021; Yu et al., 2024). Because of the rapid progress of nanomaterials applications in multiple fields, including biomaterials and biosensors, where they are applied as transducer elements and for signal amplification purposes, giving better sensor performance and reduced LOD (Qu et al., 2023; Chen et al., 2024). Gold nanoparticles (AuNPs), carbon-based nanoparticles, and quantum dots (QDs) are some of the most commonly studied categories of nanoparticles that are applied for various applications because of their unique properties (Wen et al., 2024; Chen et al., 2026).

AuNPs, in particular, have optical and electrical properties and hence are easy to synthesize and modify for biosensing applications (Balci et al., 2021). The optical properties of AuNPs allow for resonant surface plasmons upon exposure to different wavelengths of light. The oscillation of electrons does not allow their propagation through the surface, unlike the traditional SPR setups, especially in cases where the particle size is significantly less than the incident wavelength (Tai et al., 2022). QDs are semiconducting nanocrystals with luminous properties and are also applied to develop highly sensitive and selective biosensors. They have Förster resonance energy transfer (FRET) properties, which allow them to act as fluorescent quenchers, aiding in specific signal generation (Guo and Cui, 2021). Similarly, bioluminescence resonance energy transfer and chemiluminescence resonance energy transfer are similar signal generation techniques of QDs that are promising in biosensing applications (Samanta and Medintz, 2020). Carbon nanostructures like carbon nanotubes and graphene have been widely applied as electronic and electrochemical transduction systems in sensing devices (Britto et al., 2024). Carbon-based nanomaterials have unique advantages, including easy functionalization capability, high biocompatibility, and electrical properties that make them promising materials for electrochemical biosensing applications (Li et al., 2019; Nguyen et al., 2023).

#### 5.3.1 Oxidative stress markers detection

Lipid peroxidation product, 4-hydroxy-2-nonenal (4-HNE), is a toxic reactive oxidative stress marker that can be potentially detected using highly sensitive biosensors. In a recent study by Wang et al., a localized SPR immunosensor was developed for direct detection of 4-HNE in serum samples with high sensitivity and selectivity (Wang F. et al., 2024). The proposed sensor showed a low LOD of 0.15 nmol/L and a wide linear range between 1 and 20 nmol/L with high reproducibility. It can thus be applied for lipid peroxidation biomarker detection from biological samples.

Another critical oxidation byproduct that gives a condition of oxidative stress is malondialdehyde (MDA). A recent study by Bencivenga and team developed a plasmonic optical fiber immunosensor that can be potentially applied as an inexpensive POC and can detect MDA at low concentrations in saliva samples within a short period. They thus can be used as POCs under clinical settings (Bencivenga et al., 2023). Another study by Attig et al. developed a screen-printed carbon electrode functionalized with magnetic graphene oxide that could sensitively detect MDA in serum samples (Ben Attig et al., 2023). The magnetic properties of the graphene oxide allowed for easy sample preprocessing for the selective detection of MDA. The proposed biosensor worked under a broad linear range of 0.01–100 µM and could selectively detect MDA at concentrations as low as 0.010 µM. Therefore, nanomaterial-based biosensors are promising for detecting very low concentrations of oxidative stress markers that aid in sensitively detecting NASH/MASH biomarkers for early diagnosis and prognosis of liver-associated diseases.

### 5.4 Microfluidics and lab-on-a-chip platforms

Microfluidics comprises biosensing components over microfluidic chips such that the detection system is integrated with fluid infusion, and a control system that modulates signal production and transduction (Wang et al., 2025). Lab-on-a-chip platform is another terminology used to describe a miniaturized chip that is capable of autonomously carrying out biochemical processes for rapid biosensing (Arshad et al., 2023). Because of the unique design concept and fluid dynamics, microfluidic chips have several distinct advantages including microfluidic fluid flow based on the sample viscosity and allow for laminar flow through the different micropipelines. Furthermore, because of the microscale of the system, a lesser amount of reagents is required, therefore making the system more cost-effective and reducing unnecessary waste emission (Wang et al., 2025). A microfluidic biosensing system is thus a sample-to-result platform that carries out sample collection, preparation, separation, processing, and signal analysis using the same system.

#### 5.4.1 Integration with liquid biopsy approaches

As discussed in the previous sections, biopsy is a commonly used technique to assess the progress of NASH/MASH in affected populations, which is traditionally performed through surgical methods. Liquid biopsies, on the other hand, are non-invasive and allow for assessing various biomarkers in other biofluids like blood, serum, saliva, urine, and others (Connal et al., 2023). Microfluidics, owing to its unique advantages such as parallel high-throughput processing, easy functioning, precise control of reagents flow, and inexpensive availability, makes it a promising method that can be applied in liquid biopsies, especially for analyte isolation and quantitative biosensing of the biomarkers with high selectivity and sensitivity (Shi et al., 2024). Liquid biopsy integrated with microfluidics will allow for biomarker processing with high purity, enhanced recovery, and high throughput, along with highly sensitive and selective detection of the target analyte. For successful optimization in microfluidics design and operation, especially for liquid biopsy applications, the complexity in channel designs and fluidic manipulation must be standardized to overcome reliance on on-chip functions and be applied under clinical settings. Therefore, with microfluidics integration, the efficiency of personalized precision medicine, especially for NASH/MASH, can be potentially improved.

#### 5.4.2 High-throughput detection of multiple biomarkers

A significant advantage of microfluidics is running multiplexed assays in parallel. Microchannels and valves can route the samples to many detection zones and droplet systems that can encapsulate several reactions simultaneously (Convery and Gadegaard, 2019). Hence, multiple metabolites can be detected from the same sample. Multiplexed biosensors that are capable of detecting numerous biomarkers of the same type using the same systems provide for a centralized analysis system that can record the changes in biomarkers involved in similar biochemical pathways, which in turn enhances the diagnosis and sensitivity of the NASH/MASH disease progression in real time and allows for the timely assessment of treatment efficiency (Kaci et al., 2023).

However, for their successful application, a few shortcomings still need to be addressed. For starters, sample preparation is a complex process that involves different types of biomolecules that can cause interference with the biosensors. To overcome this, microfluidic sample preparation steps, like on-chip lysis, filtration, and extraction, for instance, by incorporating paper extraction pads, are being actively explored to overcome these shortcomings (Yin et al., 2019). In addition, including automation steps like cleanup post-reaction assays can also help reduce the hands-on steps and sample loss associated with it, thus enhancing the throughput. Another challenge is including heterogeneous detection methods within a standard microfluidic backend while maintaining sensitivity and avoiding crosstalk. Therefore, further research into new chip material substrates that can aid in developing inexpensive and disposable devices can help address these issues. Wearable microfluidic patches for continuous metabolic monitoring, along with artificial intelligence and cloud connectivity, can aid in developing POC devices that allow for automated image analysis and electrochemical signal processing with ease, thus aiding in reliable monitoring of NASH/MASH biomarkers.

### 5.5 Wearable and implantable biosensors

The introduction of wearable and implantable sensing technologies has revolutionized the biosensing field, bringing in a new period of closed-loop therapeutic systems. These systems allow for real-time monitoring, thus providing continuous data about the different physiological variables. This allows for a shift from traditional treatment options to better adaptive and more dynamic clinical interventions. Integrating these biosensors with therapeutics pushes the frontiers of personalized medicine by introducing drug delivery platforms like microneedles. These implantable and ingestible sensors can modulate the application of therapeutics with reference to real-time biomarker profiling.

#### 5.5.1 Metabolic syndrome monitoring

**FIGURE 5:**
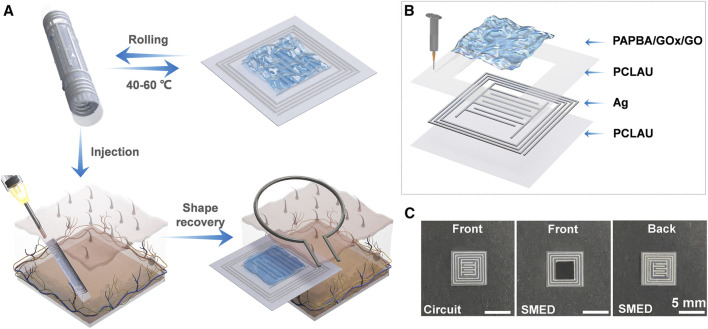
**(A)** Schematic illustration of injectable implementation of SMED. **(B)** The SMED consisting of a substrate layer (PCLAU), a circuit layer (Ag), a protection layer (PCLAU), and a sensing material layer (PAPBA/GOx/GO). **(C)** Printer circuit and fabricated device Scale bar: 5 mm.

Metabolic syndrome is a crucial risk parameter associated with NASH/MASH that includes health conditions like obesity, high blood pressure, diabetes, and elevated triglyceride levels, which all contribute towards fat buildup and inflammation in the liver that worsens the liver disease condition (Kanda et al., 2020). With the development and application of wearable and implantable biosensors, a real-time, comprehensive overview of the individual’s health can be evaluated, thus allowing for early detection and timely medical intervention. Multiple such studies have been conducted in recent years that can perform sensitive detection of metabolic syndrome-associated biomarkers using wearable and implantable biosensors. In a study by Ollmar and colleagues, a self-autonomous implantable biosensor was fabricated that could perform sensitive continuous glucose monitoring via bioimpedance measurements (Ollmar et al., 2023). The researchers proposed that the glucose sensor could be externally powered and hence could function for a long time without compromising the reliability. In another study by Jiang et al., another implantable wireless glucose biosensor was developed based on a shape memory electronic device (Jiang et al., 2023), as shown in Figure 5. The system was created using an inductive capacitive circuit alongside a biosensing coating comprising poly (3-aminophenylboronic acid), glucose oxidase, and graphene oxide over a poly (D, L lactide co caprolactone) layer that had shape memory. The proposed biosensor gave a reliable and accurate glucose biosensing response and, therefore, can be applied as a promising implantable biosensor for continuous monitoring of different metabolic syndrome-associated biomarkers.

Hence, portable biosensors like wearable ones can play a crucial role in healthcare by allowing constant tracking, diagnosis, and therapeutics. With further work in nanobiotechnology, flexible electronics, and biocompatible substrates, highly selective and user-friendly wearable sensors can be developed. Furthermore, integrating such sensors with digital healthcare platforms can provide better clinical management for individuals’ health, and aid in facilitating remote monitoring, and early treatment can be provided, thus bettering the medical care.

#### 5.5.2 Potential to adapt existing biosensors for liver disease monitoring

Existing biosensors can be further developed to fabricate highly sensitive wearable-based biosensors to target specific metabolites like cytokines using flexible electrode systems. For instance, as discussed in the previous sections, ALT/AST biosensors are being developed to create lateral flow and chip-based assays; similarly, aptamer coatings can be done over flexible patches to sense such biomarkers in transdermal fluids. In addition, microneedles or pores can be integrated over wearable sensors to conduct interstitial fluid sampling under physiological conditions. A recent example of a built-in adhesive on a skin impedance patch was developed that also included deep learning analysis to carry out non-invasive monitoring of early-stage fatty liver (Wang K. et al., 2024). The proposed system could record the changes in skin liver electrical impedance signatures associated with lipid accumulation. The proposed system, combined with wearable electronics and artificial intelligence, could be extended to monitor the NASH/MASH condition. Moreover, sweat contains rich information on various metabolites, although typically present in very low concentrations, and is also influenced by sweat rate and glandular transport dynamics (Baker, 2019). However, it is essential to note that cytokine concentrations in eccrine sweat often exhibit weak correlations and temporal delays relative to circulating levels and hepatic inflammation due to diffusion and dilution effects (Cinca-Morros et al., 2024). However, available translational and pilot clinical studies suggest that sweat cytokine profiles may track systemic inflammatory trends over time, with improved associations observed when longitudinal or trend-based analyses are compared against imaging-derived liver stiffness or biochemical markers, rather than single time-point histological grades. Thus, sweat-based cytokine sensing should be viewed as an adjunctive, non-invasive monitoring approach for longitudinal risk stratification and disease activity tracking between clinical visits, rather than a standalone diagnostic tool.

Wearable electrochemical and optical sweat biosensors have been fabricated that can continuously measure sodium (Noor et al., 2025), potassium (Jalal et al., 2024), lactate (Yang et al., 2024), and cortisol (Wang C. et al., 2024) levels. With integration with aptamer or antibody-based biosensors, other NASH/MASH-relevant biomarkers like inflammatory cytokines can be tracked. Continuous glucose monitors are available, like the Eversense glucose sensors that can be used even as a month-long implant [127]. Similar small electrodes can also be implanted near the liver or in the subcutaneous tissues to monitor the biomarker levels continuously. For instance, the microfabricated biosensors available for lactate (Shitanda et al., 2023) and ketones (Li et al., 2005) can also be adapted for ALT/AST monitoring by integrating appropriate enzyme reactions. In addition, ingestible capsule sensors or injectable hydrogel biosensors could also be applied to assess the gut-liver axis (Jung et al., 2020). However, such implantable biosensors face unique challenges like biofouling and immune response that can rapidly deteriorate their performance. Thus, antifouling coatings for such biosensors must also be explored and developed to improve the longevity and functioning of implantable NASH/MASH biosensors (Yang et al., 2023; Arshad et al., 2025).

## 6 Target biomarkers for biosensor development

**FIGURE 6:**
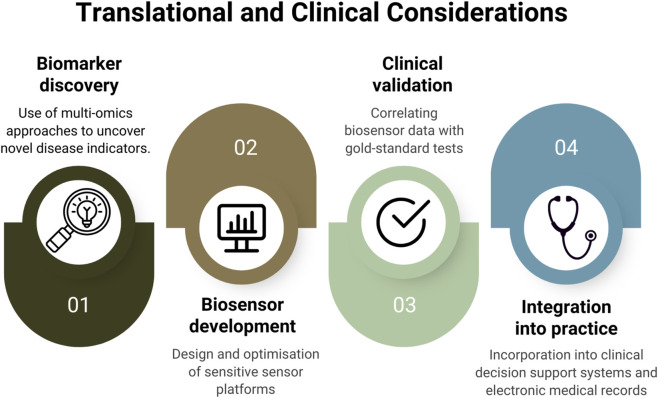
Workflow illustrating the translational pathway from biomarker discovery to clinical implementation of NASH/MASH biosensors.

For developing reliable biosensors for rapid and reliable monitoring and early intervention of liver-associated disease conditions, further work is also needed in understanding and exploring the various biomarker targets associated with NASH/MASH conditions, so that selective and specific biosensors can be further explored, thereby expanding the frontiers of biosensing and its applications (Figure 6). Table 3 provides a brief overview of possible NASH/MASH candidate biomarkers that can be applied for biosensor detection.

**TABLE 3:** NASH/MASH biomarkers that can be detected using biosensors.

Biomarker	Source	Relevance in NASH/MASH	Current detection methods
Cytokeratin-18	Serum, plasma	Marker of hepatocyte apoptosis and necrosis; differentiates NASH from simple steatosis	ELISA, electrochemical immunosensor, SPR-based immunosensor
ALT	Serum	Reflects hepatocellular injury; elevated in NASH and correlates with inflammation	Enzymatic assays, electrochemical biosensors
AST	Serum	Indicator of hepatocellular damage and fibrosis progression	Biochemical assays, amperometric biosensors
miR-122, miR-34a, miR-192	Serum, plasma, exosomes	Liver-specific microRNAs; early indicators of hepatocyte injury and fibrosis	qRT-PCR, CRISPR/Cas-based biosensors, nanopore sensors
IL-6 and TNF-α	Serum, plasma	Pro-inflammatory cytokines contributing to hepatic inflammation and fibrosis	Multiplex immunoassays, electrochemical immunosensors
Lipid peroxidation products (MDA, 4-HNE)	Serum	Indicators of oxidative stress and lipid damage in hepatocytes	Spectrophotometric assays, optical biosensors
Fibrosis markers	Serum	Indicate extracellular matrix remodeling and fibrogenesis	ELISA, SPR-based immunosensors, electrochemical biosensors

### 6.1 Serum proteins

Different protein markers identify various stages of NASH/MASH-associated liver pathology. Even though a single protein is insufficient to confirm NASH, multiple such biomarkers, especially protein markers, can be considered and explored to improve the diagnostic precision of biosensors. Commonly evaluated NASH/MASH-associated biomarkers include the inflammatory proteins like C-reactive protein and cytokeratin-18 (CK-18) fragments that are linked to liver inflammation and injury (Kanda et al., 2018). These protein markers are found in hepatocytes and indicate liver apoptosis and necrosis, and are elevated in NASH/MASH cases compared to normal individuals (Cao et al., 2013). Any change in CK-18 protein fragments levels can be applied as a tool to evaluate the effectiveness of treatments, as they decrease when better liver activity occurs, and thus making them a valuable protein marker for clinical diagnostics and therapeutics (Fierbinteanu-Braticevici et al., 2010). Other biomarkers also include ferritin (Xia et al., 2023), which is an indicator of liver fibrosis especially when linked with other markers like thrombospondin-2 (THBS2) (Kozumi et al., 2021), growth differentiation factor 15 (GDF15) (Kim et al., 2018), SELE, and IGFBP7 (Rodrigues et al., 2022; Wang et al., 2023) that in combination with clinical factors like patient age, diabetes, hypertension, BMI, gives an idea of the NASH/MASH risk stratification.

Adipokines are also protein markers that can be considered for assessing the NASH/MASH progression. These are polypeptides that are released by adipose cells and are associated with NASH-linked cirrhosis (Polyzos et al., 2009). Certain adipokines like resistin (Shen et al., 2014), leptin (Martínez-Uña et al., 2020), and adiponectin (Barbalho et al., 2023) result in inflammation and fat accumulation that drive NASH/MASH progression and also help in distinguishing fatty liver and NASH/MASH condition, thereby aiding in monitoring the disease progression. Inflammatory cytokines like IL-6 and TNF-α also serve as crucial NASH/MASH biomarkers and are correlated with disease severity, hepatic inflammation, and fibrosis progression (Duan et al., 2022).

### 6.2 Metabolites

Metabolomic studies of NASH/MASH demonstrated changes in metabolite levels, including amino acids, bile acids, free fatty acids, oxidative stress products, carbohydrate, and lipid levels (McGlinchey et al., 2022). These also include elevation in branched chain amino acids, glycocholate, phosphatidylcholine, and reduction in long chain fatty acids, cysteine, and glutathione (Tan et al., 2024). Amongst these, bile acids like glycocholate and taurocholate are significantly elevated in patients with NASH/MASH. Other metabolites, like oxidative stress markers that are produced during liver cell damage, also serve as promising NASH/MASH-associated biomarkers, including oxidized lipids, malondialdehyde (MDA), 4-hydroxynonenal (4-HNE), protein carbonyls, isoprostanes, nitrotyrosine, and others (Gabbia et al., 2021). These are a consequence of an imbalance in ROS that overwhelms the antioxidant defense of the liver, causing cellular damage leading to fibrosis and cirrhosis (Ore and Akinloye, 2019). ROS can also damage proteins, giving protein carbonyls, therefore confirming protein damage and loss of function (Radman, 2020). Isoprostanes are other biologically active compounds that result from fatty acid oxidation and serve as crucial markers of lipid peroxidation (Bauer et al., 2014). Similarly, nitrotyrosine is formed when ROS reacts with tyrosine amino acids, resulting in damage (Radi, 2013). These oxidative stress markers contribute to mitochondrial dysfunction, damage to the liver antioxidant defenses, increased endoplasmic reticulum stress, and an inflammatory cascade that involves cytokines and activation of immune cells, which further adds to oxidative stress (Allameh et al., 2023).

### 6.3 Nucleic acids

Multiple nucleic acid biomarkers are recorded to be associated with NASH/MASH, including miRNAs and long non-coding RNAs (lncRNAs) that vary in individuals with liver illnesses. Primarily, miRNAs are non-coding RNAs with ∼22 nucleotides that bind with target mRNA, pausing the translation process or resulting in mRNA degradation (Kim et al., 2021). Markers like miR-122, miR-192, and miR-34a are noted to be constantly upregulated (Hochreuter et al., 2022) in NASH patients alongside lncRNAs like Lexis and RP11-128N14.5 (Zeng et al., 2023). Elevation in these markers indicates the steatosis progress, ballooning, inflammation, and fibrosis (Turchinovich et al., 2018). It hence helps differentiate between patients with NASH/MASH as opposed to individuals affected with simple steatosis.

### 6.4 Exosomal signatures

Extracellular vesicles like exosomes are tiny lipid membranous structures that carry proteins and miRNAs, play a crucial role in cellular communication, and contribute towards disease development (Wang Q. et al., 2025). Exosomal markers like miRNAs, frizzled class receptor 7 (FZD7), and C-X-C motif chemokine ligand 10 (CXCL10) are secreted from hepatocytes and serve as an indication of liver disease development (Gan et al., 2025). FZD7 is a Wnt signaling pathway marker that is delivered through plasma-derived exosomes and serves as a promising biomarker for NASH/MASH diagnosis (Scavo et al., 2022). Similarly, CXCL10 is another exosomal signature that is also a macrophage chemoattractant and is secreted by lipotoxic hepatocytes, which gives a direct indication of MASH progress (Boonkaew et al., 2025).

## 7 Translational and clinical considerations

Despite the promise of highly sensitive NASH/MASH biosensors, several translational hurdles must be overcome. Many current biosensors rely on labile biological elements, which complicate large-scale manufacturing and shelf-life. For instance, an enzyme-based cholesterol sensor retained only ∼60% of its initial activity after 7 days of storage (Tsai et al., 2008), and a reusable phosphate sensor exhibited just ∼7% variation over 30 days (Upadhyay and Verma, 2015). Clinical samples introduce additional challenges. Non-target analogues and complex matrix effects can skew the readings; sensors validated under ideal lab conditions may perform poorly in heterogeneous clinical specimens (Chiu et al., 2010). To address this, strict standardization and quality control are essential. International medical-device standards like IEC 62304/60601, ISO 14971/13485, and rigorous calibration protocols should be applied during design, production, and testing to ensure consistency (Ravizza et al., 2019). Likewise, nanomaterial-based sensors require uniform synthesis and precise control, and hence many carbon-nanomaterial-based platforms have been noted to have limited long-term stability and reproducibility (Meskher et al., 2022; Şak et al., 2025). Thus, nanomaterial fabrication steps like synthesis and handling must be optimized to avoid batch-to-batch variability and to improve device stability. Analytical validation challenges and readiness levels of different recently explored biosensors for the detection of various liver disease biomarkers have been summarized in Table 4.

**TABLE 4:** Summary of analytical validation challenges and readiness levels of different recently explored biosensors for the detection of various liver disease biomarkers.

Platform	Target analytes	Validation challenges	Technology readiness level (TRL)	Ref.
Electrochemical transducers	Enzyme-coated electrodes for ALT/AST; aptamer/antibody sensors for fibrosis markers	1. Enzyme decay and sensor aging may cause baseline shifts requiring frequent recalibration. 2. Batch-to-batch variability of electrodes and reagents may lead to high sensor-to-sensor error. 3. In real samples, proteins and cells can sometimes foul electrodes, degrading performance.	TRL 3/4 (bench-scale prototypes)	Quan et al. (2024), Zimmerpeacock (2025)
Fiber-optic LSPR biosensors	ALT	1. Complex fabrication process. 2. Nanoparticle or biocatalyst layers must remain stable over time; optical alignment is delicate. 3. Potential cross-reactivity can produce spurious LSPR shifts, requiring careful selectivity.	TRL ∼3 (proof-of-concept)	Jamal et al. (2009), Yadav et al. (2022), Samy et al. (2023), Zimmerpeacock (2025)
Microfluidic paper-based (μPAD)	ALT, AST, albumin	1. Multiple colorimetric assays on one chip risk can result in visual response overlaps; precise reagent deposition and timing are critical. 2. Lighting, temperature, and humidity can alter color intensity and interpretation. 3. Whole blood constituents like cells, hemoglobin, and variable hematocrit can skew results; they require a filter or preprocessing layers.	TRL 4/5 (pilot studies)	Pollock et al. (2012), Yang et al. (2018), Chinnappan et al. (2023), Zimmerpeacock (2025)
SERS (Raman) biosensors	Bilirubin	1. Fabricating uniform nanostructures can be difficult; batch-to-batch variability affects sensitivity. 2. Serum fluorescence and other biomolecules can obscure target signals; this requires a sample enrichment or preconcentration step. 3. SERS intensity can vary nonlinearly; it requires careful calibration and multivariate analysis of spectra.	TRL ∼3 (lab prototypes)	Liang et al. (2021), Zimmerpeacock (2025)
Molecularly imprinted polymer (MIP)	HSA	1. Large flexible proteins are complex to imprint; binding sites may not perfectly match the albumin conformation. 2. Incomplete extraction of the albumin template can reduce binding capacity and specificity. 3. Binding to polymer sites is often slower and weaker than antibody-based capture, resulting in higher LOD. 4. Polymer swelling and stability issues may occur.	TRL ∼3 (research stage)	Akgönüllü et al. (2023), Zimmerpeacock (2025)

Validation must also extend beyond the bench to confirm consistent performance across settings and populations. Multi-center and multi-operator studies are needed to verify that a given biosensor yields the same output across different laboratories and patient cohorts. For instance, the V3 framework of the Digital Medicine Society distinguishes verification of raw data acquisition from analytical validation of the signal-processing algorithms (Goldsack et al., 2020). This implies that bench tests and *in vivo* studies should demonstrate that the embedded algorithms reliably convert raw biosensor signals into accurate physiological metrics. In addition, clinical validation against gold-standard endpoints like new biosensor measurements should be compared to biopsy-proven fibrosis scores or imaging results in large, diverse cohorts. NAFLD/NASH affects subgroups differently by age, sex, ethnicity, genetics, and co-morbidities; therefore, validation studies must be sufficiently powered and inclusive.

More importantly, given the volume of data from continuous biosensors, artificial intelligence and machine learning can be integrated to extract clinically actionable information. AI models can identify subtle temporal patterns like small biomarker trends that precede overt disease changes. In pathology, AI-based tools such as the FDA-qualified AIM-NASH system (US Food and Drug Administration, 2025) are already being used to analyze liver biopsy images and automatically score features like steatosis, inflammation, and fibrosis stage. A similar approach can be potentially explored for biosensor data. An algorithm could be developed that could possibly “score” the longitudinal metabolic profile of a patient to estimate fibrosis progression or treatment response. In addition, biosensor-enabled care could be integrated with telemedicine. Patients with early-stage NAFLD could wear a home biosensor that continuously transmits data to a cloud platform. Clinicians could then review live biomarker trends during virtual visits and adjust management in real time. Remote monitoring has already shown concrete benefits in other conditions, like a study involving a home telemonitoring trial for high-risk patients demonstrated significant reductions in hospital readmissions and emergency visits (Po et al., 2024). Similarly, continuous glucose monitors (CGMs) in diabetes care enable patients to tightly control blood sugar without frequent clinic visits. For instance, Dexcom reports that its G7 sensor with 15-day wear and 8.0% MARD accuracy is the longest-lasting and most accurate CGM system (Dexcom, 2018; Dexcom, 2025). By analogy, a validated liver biosensor could allow hepatologists to manage more patients virtually, with sensor data feeding clinical decision support tools in the electronic health record.

However, equivalent POC or wearable sensors for liver-specific markers are still limited. So far, only two FDA-cleared devices, Reflotron Plus (Roche) and Cholestech LDX (Alere), are available that can measure ALT/AST from a fingerstick without lab separation (Li and Cheng, 2024). Several research groups are working on paper-based or microfluidic transaminase tests (Chinnappan et al., 2023). Once validated, such liver enzyme biosensors could transform NAFLD/NASH care by enabling immediate, at-home screening. Patients at risk could do a fingerstick ALT/AST test at home, rather than awaiting central lab results. This would allow clinicians to catch liver injury earlier and intervene sooner in the metabolic dysfunction pathway of NAFLD/NASH.

## 8 Future directions

**FIGURE 7:**
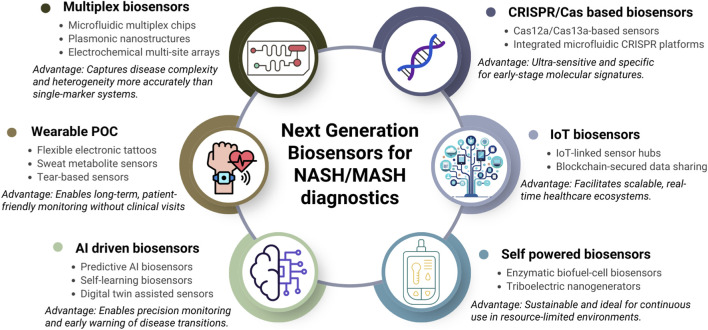
Possible next-generation biosensors that have promising potential in NASH/MASH diagnostics.

As discussed in the previous sections, NASH/MASH diagnosis and monitoring currently rely on invasive biopsy methods or indirect blood tests, which call for less invasive or non-invasive monitoring tools like biosensors. Biosensors for detecting liver disease biomarkers remain largely at an experimental stage, partly because of the clinical validation and standardization procedures. This gap suggests that two similar studies proposed for the same biomarker can yield inconsistent results owing to multiple factors, including the difference in environmental factors, possible handling error, variation in laboratory conditions, and minor differences in experimental protocols, thus making it hard to compare studies or establish clinical cutoffs. Furthermore, sample matrix effects are another factor. For instance, though ALT/AST levels are typically measured in serum, many studies have also explored devices that can sample the biomarkers in interstitial fluids or sweat (Davis et al., 2024; Zhyvolozhnyi et al., 2024). Thus, a biosensor calibrated in buffer or serum may not be able to detect the biomarkers in other biofluids accurately. With further work, emerging biosensor technologies aim to fill this gap by measuring various NASH-associated biomarkers in real-time. The proposed next generation sensing methods will be a promising biosensors device for accurate diagnosis of NASH/MASH (Figure 7).

### 8.1 Multi-analyte biosensing

Complex diseases like NASH involve multiple pathways, so assessing a single analyte may not provide the complete picture. With the advent of multi-analyte platforms that measure different classes of biomarkers simultaneously, for instance, monitoring proteins like CK-18 fragments, IL-6, metabolites like glucose, lipids, and circulating miRNAs from a single sample can help provide a more comprehensive metabolic profile (Chu et al., 2022; Klebes et al., 2024). For instance, microfluidic lab-on-a-chip devices and organic electrochemical transistors can be engineered with multiple sensing gates or electrodes such that each is functionalized for a different target. In a recent study, an organic electrochemical transistor-based sensor array was developed to detect 3 different metabolites, including uric acid, cholesterol, and glucose in saliva samples, simultaneously with low LOD in the nanomolar range (Lu et al., 2025). Multiplexed electrochemical sensor arrays have also been built, like printed paper chips to detect proteins like C-reactive protein (CRP), troponin I (cTnI), and procalcitonin (PCT) in parallel (Boonkaew et al., 2021), and a 3D printed amperometric biosensor could quantify cholesterol and choline at the same time (Koukouviti and Kokkinos, 2021). These examples, therefore, illustrate how multiple enzymes, antibodies, and aptamers can be combined over a single platform to allow for a broad metabolic profiling within a small sample. Thus, lab-on-chip prototypes combining immunoassay and nucleic acid amplification steps could be adapted for promising NASH/MASH monitoring.

### 8.2 Wearable POC devices

Another emerging direction is the wearable and smartphone-based biosensors for remote monitoring that do not require regular clinical visits. Such patches can detect a range of analytes, including electrolytes, stress hormones, and cytokines, with minimal user effort. Notably, IL-6, a key inflammatory marker, has been measured in sweat by a wearable immunosensor (Gao et al., 2023). In a recent clinical pilot, cirrhosis patients wore a sweat sensor (Sweat AWARE) that continuously tracked IL-6, C-reactive protein, and TNF-α. The results showed that the sweat measurements correlated with blood levels and distinguished patients from controls (Davis et al., 2024). This proof-of-concept shows that sweat-based monitoring of liver inflammation is feasible and thus is a promising noninvasive continuous monitoring system for liver disease management. Therefore, in the future, similar patches could be used at home to monitor inflammatory status or metabolic response in NASH patients.

### 8.3 AI-driven analysis

Advanced machine learning tools can be applied to complex, high-dimensional biosensor outputs to recognize patterns of inflammation or fibrosis and improve diagnostic accuracy in NASH/MASH patients (Lu et al., 2025). ML algorithms can analyze high-dimensional biosensor outputs, identify subtle trends, and classify disease states more accurately than simple thresholding. For instance, neural networks or random forest models (Sinha et al., 2023) can be trained on multi-biomarker signatures to distinguish active NASH from stable NAFLD, or to predict fibrotic progression. Other possible applications include training classifiers on continuous IL-6/CRP sweat profiles to predict impending complications, using deep learning to analyze multiplex sensor arrays, or applying unsupervised learning to discover new biomarker combinations from multi-omics sensor data. Such an application was observed in a recent study, where continuous sweat measurements in cirrhosis revealed circadian patterns of IL-6 and CRP levels that rose in the evening and fell overnight (Davis et al., 2024). An AI system could detect such temporal patterns automatically, triggering alerts when an aberrant inflammatory signature was observed. Similarly, ML used in other hepatic contexts, like evaluating histology images (Taylor-Weiner et al., 2021), can be applied to enhance NASH detection, thus suggesting that these methods can readily extend to biosensor data. Furthermore, integrating biosensors with cloud-based analytics and ML pipelines could yield smart monitoring systems that learn patient baselines and alert clinicians to meaningful deviations, therefore allowing for early medical intervention.

## 9 Conclusion

Despite the growing burden of NASH/MASH, current diagnostics remain suboptimal. Definitive diagnosis still relies on liver biopsy, which is an invasive, costly procedure with sampling risk. On the other hand, the current noninvasive imaging or blood tests provide incomplete information with reference to the patient’s health and disease progression (Tantu et al., 2025). These limitations leave many cases undetected until late stages. Therefore, there is a clear, rising, unmet need for dynamic, accessible monitoring tools to identify metabolic injury before irreversible fibrosis sets in.

Biosensors offer a promising path to fill this gap. Advanced biosensing platforms can continuously track key vital signs and biochemical signals, turning these into early warning indicators of hepatic stress. Paired with AI-driven analytics, such devices enable precise, noninvasive surveillance of a patient’s metabolic state. They can also indicate subtle deviations long before critical biomarkers rise (Yammouri and Ait Lahcen, 2024). Similarly, emerging point-of-care biosensors can measure multiple analytes simultaneously, including genetic markers, inflammatory cytokines, and fibrosis-related proteins in real time (Tantu et al., 2025). Realizing this vision will require a concerted and interdisciplinary effort. There is a need for hepatologists and clinical researchers to work closely with bioengineers and data scientists to identify the most informative biomarkers and validate sensor outputs against clinical outcomes. The potential payoff of transforming the silent liver disease that can be monitored and managed in real-time will transform the diagnostics and medical landscape. Thus, by harnessing biosensor innovation and AI, while navigating the scientific, clinical, and regulatory hurdles, the field can move toward proactive, patient-centered care for NASH/MASH.
